# Multiomics analysis of tolerant interaction of potato with potato virus Y

**DOI:** 10.1038/s41597-019-0216-1

**Published:** 2019-10-31

**Authors:** Tjaša Stare, Živa Ramšak, Maja Križnik, Kristina Gruden

**Affiliations:** 1 Department of Biotechnology and Systems Biology, National Institute of Biology, Večna pot 111, 1000 Ljubljana, Slovenia; 2 Jožef Stefan International Postgraduate School, Jamova 39, 1000 Ljubljana, Slovenia

**Keywords:** Time series, Biotic

## Abstract

Potato virus Y (PVY) is the most economically important viral pathogen of potato worldwide. Different potato cultivars react to the pathogen differently, resulting in resistant, tolerant or disease outcome of the interaction. Here we focus on tolerant interaction between potato cv. Désirée and PVY^NTN^. To capture the response in its full complexity, we analyzed the dynamic changes on multiple molecular levels, including transcriptomics, sRNAomics, degradomics, proteomics and hormonomics. The analysis was complemented by the measurements of viral accumulation, photosynthetic activity and phenotypisation of the symptoms. Besides cv. Désirée we also studied its transgenic counterpart depleted for the accumulation of salicylic acid (NahG-Désirée). This multiomics analysis provides better insights into the mechanisms leading to tolerant response of potato to viral infection and can be used as a base in further studies of plant immunity regulation.

## Abstract (summary)

**Table:** 

Measurement(s)	transcription profiling assay • small RNA sequencing assay • protein profiling assay • hormone measurement • photosynthesis
Technology Type(s)	RNA sequencing • nanoflow liquid chromatography-tandem mass spectrometry • gas chromatography-mass spectrometry • fluorometer
Factor Type(s)	days post inoculation
Sample Characteristic - Organism	Solanum tuberosum

Machine-accessible metadata file describing the reported data: 10.6084/m9.figshare.9891830

## Background & Summary

Plant pathogens are responsible for up to 30% losses in agriculture^1^. Viral caused plant diseases affect all major crops. Being obligate intracellular organisms, chemical control of these pathogens can only be applied to control their insect vectors. Marker-assisted selection breeding of plants that are tolerant or resistant to viruses is an alternative option of control. For this strategy, understanding molecular responses of plant immunity is crucial^2,3^. At the molecular level, plant defense mechanisms against viral pathogens are regulated by a network of interconnected signal transduction pathways, which lead to plant metabolism reprogramming^4^. It is also insufficient to analyze plant status snapshots in a single time point and scale, instead, dynamic changes should be monitored on multiple molecular levels to understand the underlying mechanisms^5,6^. With this in mind, we performed multiomics analyses of potato (*Solanum tuberosum* L.) responses following the infection with potato virus Y (PVY).

Potato is the world’s most important non-grain staple crop. Viruses pose a serious threat to potato production, not only because of the effects caused by the primary infection, but also because potato is propagated vegetatively and the viruses are transmitted through the tubers and accumulate over time^7^. The most devastating potato virus is PVY^8^, which induces severe symptoms in sensitive potato cultivars, including the development of potato tuber necrotic ringspot disease^9,10^. One of the most widely grown potato cultivars is cv. Désirée, which is in non-stress condition tolerant to PVY^NTN^, meaning that the virus replicates and spreads systemically, however, symptoms of the disease are reduced or not visible at all^11^. Tolerance may have an advantage over resistance for crop protection because it does not actively select against virus infection and replication, therefore there is little evolutionary pressure for PVY to mutate. Hence, the tolerant phenotype is likely to be more durable than resistance^12^.

**Fig. 1:**
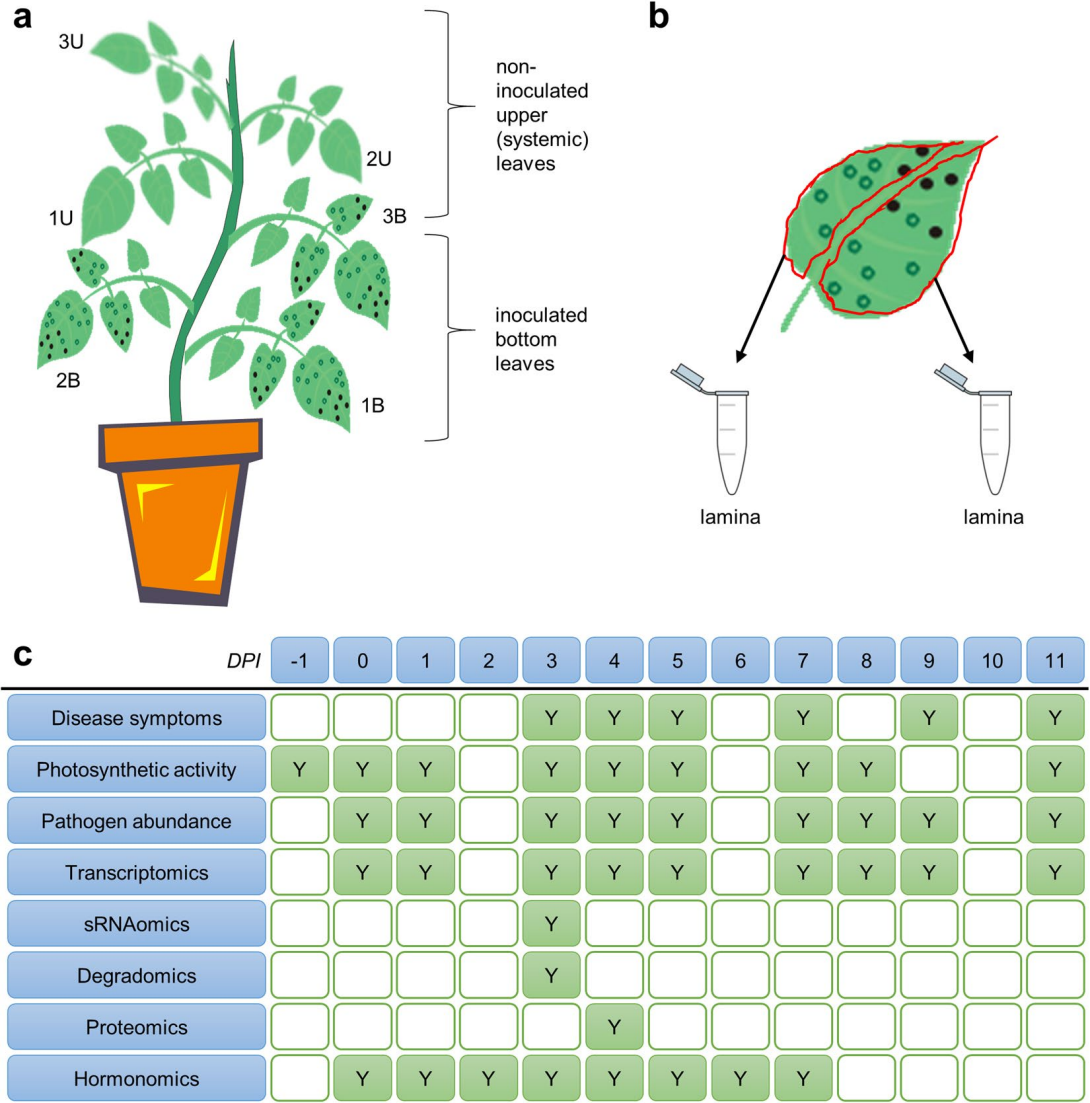
Overview of experimental design and sampling. (**a**) Plant scheme, indicating the nomenclature for leaves (1B – first bottom, 2B – second bottom, 3B – third bottom; 1U – first upper, 2U – second upper, 3U – third upper leaf). (**b**) Leaf sampling scheme, where for each leaf, two lamina samples were collected. (**c**) For each measurement method, indication of which day post infection (DPI) the samples were collected is given.

**Fig. 2:**
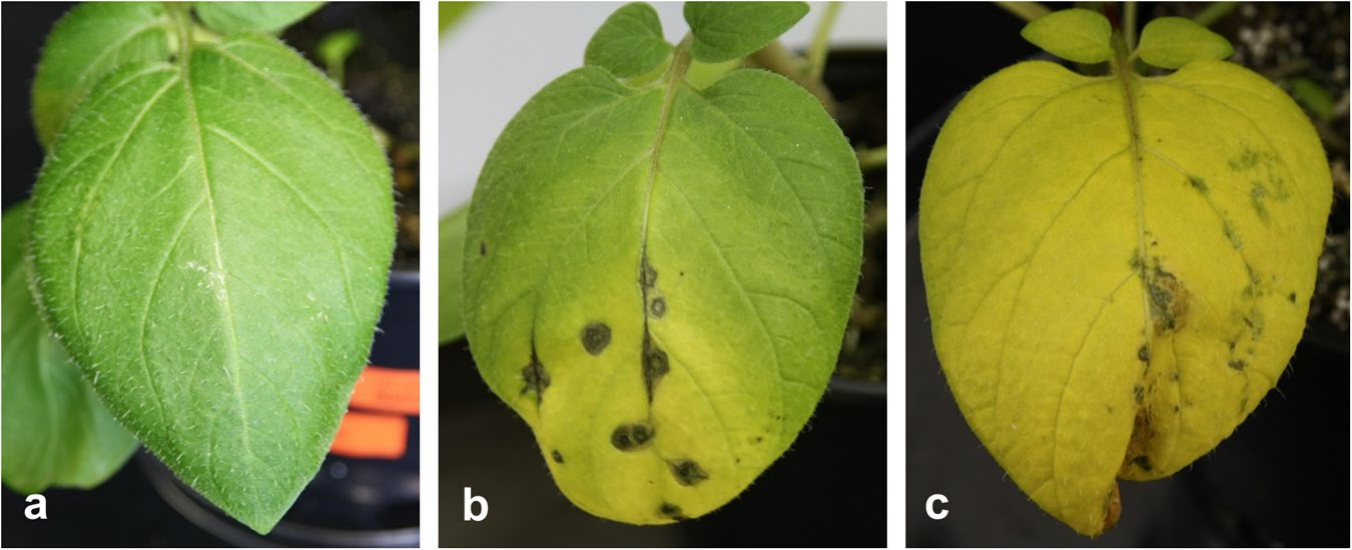
Examples of observed phenotypes in potato cv. Desiree-PVY^NTN^ interaction during the course of the experiment. (**a**) Leaf with slight mechanical damage. (**b**) Leaf exhibiting spot and vein necrosis and yellowing. (**c**) Leaf exhibiting yellowing.

In this study, we performed a comprehensive, time series multiomics analysis of the potato cv. Désirée responses to PVY^NTN^ infection^11^. The response has been analyzed on the levels of transcriptomics, sRNAomics, degradomics, proteomics and hormonomics. The analysis was complemented by the measurements of viral accumulation, measurements of photosynthetic activity and symptoms development (Figs 1 and 2). The plants were either mock- or PVY- treated and the response was analyzed in infected and systemic (upper noninoculated) leaves at different time points, one day before the infection (-1 days post inoculation (dpi)), on the day of infection (0 dpi) and on the following days after infection: 1, 2, 3, 4, 5, 6, 7, 8, 9 and 11 dpi^4,13^ (Fig. 1). As salicylic acid (SA) was found to be a crucial component for disease symptoms attenuation^14^, NahG-Désirée transgenic plants, producing salicylate hydroxylase, which catalyzes the conversion of SA to catechol^11,15,16^, were also included in experimental design (Fig. 1).

Plant growth was unaffected in this tolerant interaction. However, in the inoculated leaves virus multiplication was detected at 5 dpi onwards and virus spread to upper systemic leaves was detected at 7 dpi. Also, leaf yellowing occurred faster in virus-inoculated compared to mock-inoculated plants. SA-depletion rendered NahG-Désirée plants more susceptible to PVY^NTN^ infection, which was manifested as faster virus multiplication and appearance of strong disease symptoms including spot necroses, vein necroses and chlorotic spots beside more pronounced yellowing^13^. To evaluate dynamic changes in photosynthetic activity, net photosynthesis, stomatal conductance, actual photochemical efficiency, potential photochemical activity, chlorophyll content and electron transport rate were measured on both mock- and PVY-treated plants of both genotypes^13^. In both, a decrease in net photosynthesis and stomatal conductivity at 5 dpi was observed, which coincided with the onset of the virus accumulation. Additionally, in PVY-infected plants, a transient decrease of photochemical efficiency was observed at 5 dpi.

**Fig. 3:**
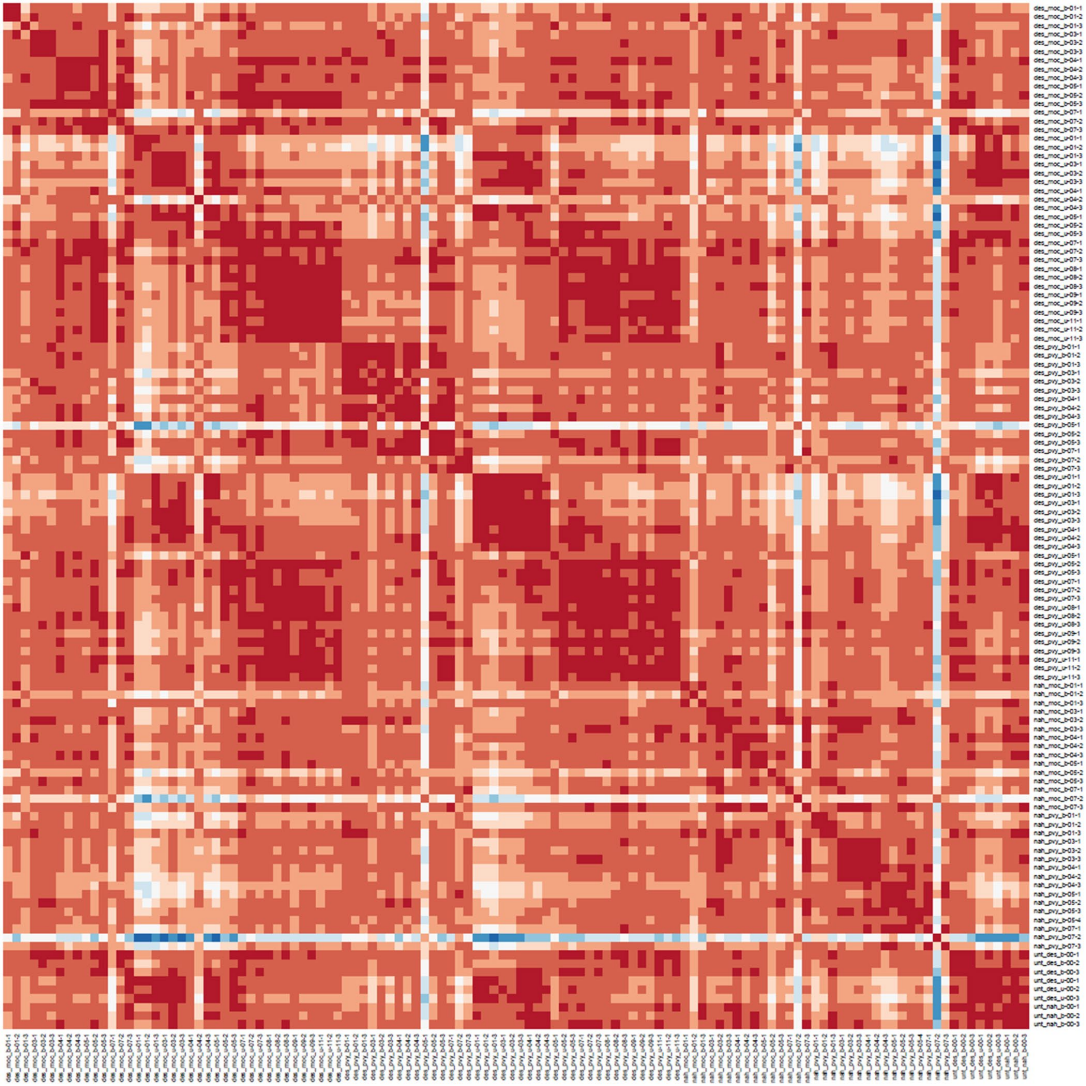
Overview of responses on the transcriptomics level (microarray data). Heatmap showing hierarchical clustering of transcriptome microarray samples using the Pearson correlation coefficient (PCC) as a distance measure, without reordering. High similarities can be observed between similar biological conditions and three biological conditions (heatmap diagonal). Actual PCC values were all higher than 0.85, even for the samples colored in blue (n = 118).

**Fig. 4:**
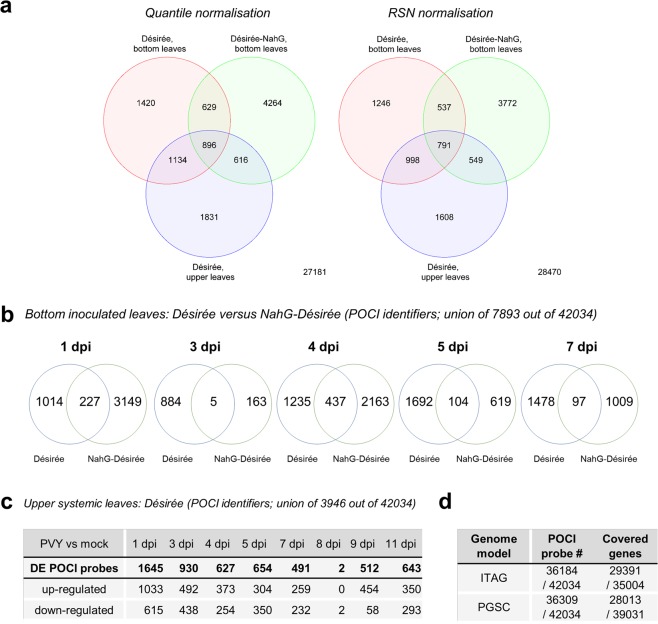
Comparison of transcriptomics responses in potato-PVY interaction through time. (**a**) Venn diagrams of differentially expressed (DE) genes in three different genotype/leaf type combinations (Désirée bottom, NahG-Désirée bottom and Désirée upper leaves) and for two normalisations (RNS and quantile). (**b**) Venn diagrams of differentially expressed (DE) genes in virus versus mock treated plants in bottom leaves of cv. Désirée and NahG-Désirée genotypes at 1, 3, 4, 5 and 7 dpi. (**c**) DE genes for upper non-inoculated leaves are shown for Désirée plants at 1, 3, 4, 5, 7, 8, 9 and 11 dpi. The values represent statistically significant differentially expressed genes as determined by empirical Bayes method (n = 3, FDR p-value < =0.05, |logFC| > =0.8). (**d**) Coverage of the two potato genome models (ITAG, PGSC) by the POCI microarray features.

Transcriptomics measurements of infected potato leaves of both genotypes for both mock- and PVY-inoculated plants were performed at 0, 1, 3, 4, 5 and 7 dpi using POCI arrays that are able to capture the expression of 84% of the predicted ITAG genes and 72% of predicted PGSC genes. To evaluate dynamics of systemic response, upper untreated leaves were also analyzed at 0, 1, 3, 4, 5, 7, 8, 9 and 11 dpi, for cv. Désirée. Overall, high similarities were observed between biological triplicates (Fig. 3). In untreated plants (0 dpi), 384 POCI probes were differentially expressed between genotypes (Désirée vs. NahG-Désirée). Infection of potato bottom leaves with PVY caused dynamic reprogramming of transcription, 3,572 POCI probes (out of 42,034) were differentially expressed over all time points in inoculated leaves of cv. Désirée and 5,649 in NahG-Désirée. The most pronounced viral induced changes occurred in Désirée plants at 5 dpi and NahG-Désirée at 1 dpi (Fig. 4b).

Plant response on the small RNA (sRNA) level was analyzed at 3 dpi in mock- and PVY-inoculated bottom leaves of both genotypes. We identified 249 previously described plant (*Viridiplantae*) microRNAs (miRNAs) belonging to 96 miRNA families. In addition, 186 novel miRNAs (from 155 *MIR* loci), 1,513 phased small interfering RNAs (phasiRNAs; from 482 *PHAS* loci) and more than 46,000 PVY-derived siRNAs (vsiRNAs) were identified. In total, 97 unique sRNAs were found to be significantly differentially expressed between mock- and PVY-infected Désirée plants. In NahG-Désirée only 28 miRNAs were differentially expressed, with the majority showing a lower degree of induction than in Désirée plants^13^. Depletion of SA thus attenuates the response of potato cv. Désirée to PVY on the sRNA level.

Degradome-Seq was performed on the samples collected 3 dpi, for this analysis biological replicates were pooled. This data complemented the *in silico* predictions for identification of sRNA target transcripts and to construct sRNA regulatory network^13^. By combining expression changes of sRNAs and their target transcripts, 92 interaction pairs with the negatively correlated expression pattern were identified^13^. In addition to previously described regulation of immune receptor transcript, a novel connection between sRNAs and gibberellin biosynthesis was discovered, linking immune and developmental signaling pathways^17^. The cumulative effect of sRNAs-mediated regulation of gibberellin biosynthesis genes was also confirmed by hormonal content measurements^13^.

Proteomics measurements, using liquid chromatography coupled to Orbitrap LTQ XL mass spectrometer, were performed in mock- and PVY-inoculated bottom leaves samples of both genotypes, collected at 4 dpi. Two approaches for peptide and protein identification and quantification were applied: 339 proteins were identified with spectral counting, and 250 with MaxQuant approach^13^. The number of identified proteins is limited as we have applied an approach that has not involved any subsampling prior to analysis thus the majority of identified spectra corresponded to Ribulose-1,5-bisphosphate carboxylase oxygenase which is by far the most prevalent protein in plant leaves (30–50%). From either approaches, 21 proteins showed significantly altered changes in their abundance in response to viral infection.

## Methods

### Plant material

Potato (*Solanum tuberosum* L.) cv. Désirée and transgenic potato plants expressing SA hydroxylase (NahG-Désirée) were propagated in tissue culture. The plantlets were re-propagated every 6 weeks until stem nodes were transferred to MS30 media for rooting. Two weeks after node segmentation, the plantlets were transferred to soil. Throughout the experiment, tissue culture-, as well as soil-grown plants were kept in a growth chamber with a 16-h photoperiod. Tissue culture growth chamber was set to 19 °C overnight temperature and 21 °C day temperature with 90% humidity in both periods. Soil growth chambers were set to 22 °C and 70% relative humidity during the day and 20 °C with 65% relative humidity overnight. The exact growth conditions during the 16-h light period were measured by a Li-6400 (LiCor, Lincoln, USA): light intensity 125 μmol m^−2^ s^−1^, temperature 25 °C, 700 µmol CO_2_ mol^−1^, and relative humidity 65–75%. After 4 weeks of growth in soil, the potato plants were inoculated with the sap of healthy (mock) or the sap of PVY^NTN^ (isolate NIB-NTN, AJ585342) infected potato cv. Pentland grown in tissue culture. Three bottom leaves were dusted with carborundum powder and rubbed with the sap. After 10 min, leaves were extensively washed with tap water.

For transcriptomics, sRNAomics, degradomics and proteomics measurements three inoculated leaves (bottom, abbreviated as B, Fig. 1) were sampled at 1, 3, 4, 5 and 7 dpi. Inoculated leaves could not be collected at later time points due to leaf-drop. The three systemic leaves (upper, abbreviated as U, Fig. 1) were sampled at 1, 3, 4, 5, 7, 8, 9 and 11 dpi. The first systemic leaf was the one directly above the first (oldest) inoculated leaf (Fig. 1). Sampling of untreated plants was done for both genotypes (Désirée, NahG-Désirée) and they were designated as untreated control (0 dpi). Additional plants were either mock- or PVY-treated and analysis of photosynthetic activity was performed from -1 dpi to 11 dpi in inoculated and systemic leaves for both genotypes. For hormonome analysis an additional set of Désirée and NahG-Désirée plants was grown. Two bottom leaves per plant were inoculated. Leaf samples from four different mock- and PVY-inoculated plants of Désirée and NahG-Désirée were collected in eight consecutive time points (0–7 dpi).

All the sample information, with corresponding MIAPPE description of the experiment is given at FAIRDOMHub under the investigation “*MOA - Multiomics analysis of potato response to Potato virus Y (PVY) infection*”^13^.

### Transcriptomic analysis

For transcriptomic analysis, we sampled the first bottom (1B) leaves of Désirée and NahG-Désirée plants (1, 3, 5, and 7 dpi) and the first upper (1U) leaves of non-transgenic Désirée (1, 3, 5, 7, 8, 9 and 11 dpi). Total RNA from the inoculated leaves was extracted, DNase treated, purified, and quality controlled as described previously^18^. A one-color based hybridization protocol was performed on the custom 60-mer oligo microarrays (4 × 44 K; AMADID 015425) designed by the Potato Oligo Chip Initiative^19^. For each sample, at least 1 μg total RNA was used and sent for analysis at IMGM Laboratories GmbH, Germany. The raw data were analyzed in R (R Development Core Team, 2011; version 2.13.2)), using the Agi4x44PreProcess^20^ and limma packages^21^.

The microarray features were filtered according to the Agilent quality control flags: if the feature was determined to be well above background (feature signal standard deviation (SD) was greater than 2.6 of it’s surrounding background SD; IsNOTWellAboveBG^20^) and if the feature was not saturated (<50% of the pixels were below the saturation threshold; IsSaturated^20^). If in at least 10% of the total microarray count (11) the feature’s flag was ok, then it was retained for further analysis. Raw data of the remaining 37.865 (from a total of 42.034) features was robust spline normalized (rsn^22^). The empirical Bayes method^23^ was used to detect differentially expressed genes between mock- and PVY-inoculated plants at each time point and for each genotype (Benjamini and Hochberg’s^24^ (FDR) adjusted p ≤ 0.05).

### sRNAomics

For sRNA analyses, second bottom inoculated leaves were sampled at 3 dpi, which corresponds to early stages of viral multiplication for both genotypes and before symptoms development in NahG-Désirée plants. Total RNA was extracted from 100 mg of homogenized leaf tissue using TRIzol reagent (Invitrogen, Carlsbad, CA, USA) and MaXtract High Density tubes (Qiagen, Hilden, Germany) following manufacturers’ protocols. RNA concentration, quality and purity were assessed using agarose gel electrophoresis and NanoDrop ND-1000 spectrophotometer (Thermo Fisher Scientific, Waltham, MA, USA). sRNA NGS libraries were generated from total RNA samples using the TailorMix miRNA Sample Preparation Kit (SeqMatic LLC, Fremont, CA, USA) and subjected for 50 bp single-end sequencing on the Illumina HiSeq 2000 Sequencing System at SeqMatic LLC.

The raw sRNA sequencing reads were first trimmed to remove adaptor sequences using CLC Genomics Workbench 8 (https://www.qiagenbioinformatics.com/products/clc-genomics-workbench) and further filtered according to quality with Filter Tool (UEA sRNA Toolkit)^25^ by discarding low complexity reads (containing at most two distinct nucleotides), reads shorter than 18 nt and longer than 25 nt, reads matching tRNA/rRNA sequences and reads not mapped to the potato genome (PGSC_DM_v4.3)^26^. To identify known annotated miRNAs, the remaining reads were compared to all plant miRNAs registered in the miRBase database (release 21^27^), allowing no mismatches. The sequences that matched mature miRNAs from other plants than potato (miRNA orthologs), were mapped to the potato genome to find corresponding *MIR* loci able to form a hairpin structure^28^ and named according to the annotation of conserved miRNA^29^. miRNAs that had different 5′ and 3′ ends with respect to the mature miRNA, were annotated as miRNA variants (isomiRs). To identify novel unannotated miRNAs, filtered reads were submitted to miRCat tool (UEA sRNA Toolkit)^25^ using default parameters for plants, considering only reads of lengths 18–24 nt. Reads were first mapped to the potato genome^26^, then the 100 and 200 nt long windows around the aligned reads were extracted^28^. The predicted secondary structures were trimmed and analyzed to verify the characteristic hairpin pre-miRNA structure according to plant miRNAs annotation criteria^29^. An additional criterion we have imposed was, that novel miRNAs should be present at least in two analyzed samples with more than five raw reads. Potential novel miRNAs were mapped against miRBase and sequences that matched known plant miRNAs with up to three mismatches were excluded. The novelty of potato specific miRNAs was verified by comparison with the miRPlant version 5^28^ using default parameters and additionally rechecked against the latest releases of Rfam^30^, tRNA^31^ and snoRNA^32^ databases. Families of novel miRNAs were determined by clustering their precursor (pre-miRNA) sequences with pre-miRNAs of annotated known miRNAs from miRBase using CD-HIT-EST^33^ with an identity threshold of 0.9. The sequences showing similarities with annotated pre-miRNAs were grouped into corresponding known miRNA family, and sequences that did not show similarity with known plant miRNAs, were classified as novel miRNA families.

Prediction of phasiRNAs and phasiRNA-producing loci (*PHAS* loci) was performed using ta-siRNA prediction tool^25,34^; utilizing the potato genome^26^ and the merged potato gene and unigene sequences StNIB_v1^35^. Analysis of phasing was performed in 21- and 24-nt intervals. To detect *PHAS* loci with a significant degree of phasing, very strict criteria were applied to avoid detection of false positives (phasing Bonferroni corrected p-value < 0.05; the number of unique phasiRNAs detected at specific *PHAS* locus ≥ 4, also to avoid detection of repeat-associated siRNAs). To identify siRNA generated from viral RNA (i.e. vsiRNAs), reads of lengths 20–24 nt from all PVY^NTN^-infected samples were mapped to the reconstructed consensus PVY^NTN^ genome^36^ using CLC Genomics Workbench version 8 (http://www.clcbio.com/) allowing only 100% identity.

Differential expression analysis was performed in R (R Development Core Team, 2011; version 3.2.2), using the limma package^21^. In short, sRNA counts with a baseline expression level of at least one reads per million of mapped reads (RPM) in at least three samples were TMM-normalized (edgeR package^37^) and analyzed using voom function^38^. To identify differentially expressed sRNAs the empirical Bayes approach was used and the resultant p-values were adjusted using the FDR method. Adjusted p-values below 0.05 were considered statistically significant.

### Quantitative real-time PCR analysis

Relative concentration of the PVY^NTN^ RNA^39^ and expression of genes encoding proteins involved in photosynthesis (RuBisCO activase (RA) and chlorophyll a-b binding protein (CAB and CAB_NEW), sugar metabolism (granule-bound starch synthase I (GBSSI), β-1,3-glucanases (GluI), (GluII), (GluIII), cell wall invertase (INV) and pathogenesis-related protein 1b (PR-1b) were analyzed using RT-qPCR. Cytochrome oxidase (cox^40^) and 18S rRNA (Eukaryotic 18S rRNA TaqMan endogenous control; Applied Biosystems, Carlsbad, CA, USA) were used as endogenous controls. Newly designed primers and probes were designed as described in^18^. Analysis was performed with the same RNA samples as for microarray analysis. DNase-treated (Invitrogen; 0.1 U/DNase per µg RNA) total RNA (1–2 µg) was reverse transcribed using High Capacity cDNA Reverse Transcription kits (Applied Biosystems) as described^18^. The samples were analyzed in the set-up for RT-qPCR as previously described^41^, in 5 µl reactions using SYBR Green or TaqMan chemistry. The details on primer and probes are given according to MIQE standards^13^. The standard curve method was used for relative gene expression quantification. The transcript accumulation of each gene was normalized to the average expression of cox and 18S rRNA^11^.

The expression level of six differentially expressed miRNAs; stu-miR390-5p, stu-miR398a-5p, stu-miR408b-5p, stu-miR4376-5p.1, stu-miR6022-3p and stu-miR827-5p was quantified in relation to the endogenous control miRNA (stu-miR167a-5p.1)^17^. TaqMan MicroRNA Assays (Thermo Fisher Scientific) were designed based on the sRNA-Seq sequence of the selected miRNAs. The details on primer and probes are given according to MIQE standards^13^. Total RNA (1 µg) of the same samples as used for sRNA-Seq was DNase I (Qiagen) treated and reverse transcribed using SuperScript III First-Strand Synthesis System and stem-loop Megaplex primer pool (both Thermo Fisher Scientific) following the manufacturer’s protocol and previously optimized cycling parameters^42^. Three different negative controls were included: no template control RT reactions (to assess potential Megaplex primer pool background), RT-minus controls (to check the presence of the signal that could be the result of contaminating DNA) and no template qPCR control reactions (to control for the contamination of the PCR reagents). All controls were negative. qPCR reactions were performed in 10 µl volume on the LightCycler480 (Roche Diagnostics Ltd., Rotkreuz, Switzerland) in duplicates and two dilutions (8- and 80-fold) per sample using TaqMan Universal Master Mix II, no UNG (Thermo Fisher Scientific) and TaqMan MicroRNA Assays following the manufacturer’s protocols. Additionally, for each miRNA assay, a standard curve was constructed from a serial dilution of the pool of all samples. Raw Cq values were calculated using the second derivative maximum method (Roche Diagnostics Ltd.) and miRNA expression was quantified using a relative standard curve method by normalization to the endogenous control using quantGenius^43^. The statistical significance was assessed by Student t-test.

### sRNA target prediction, degradomics and regulatory network construction

*In silico* identification of potato transcripts targeted by identified sRNAs was performed using the psRNATarget^44^, and StNIB_v1^35^ sequences as a reference, following previously proposed stringent parameters^45^. Targets of identified sRNAs were experimentally validated with a parallel analysis of RNA ends (PARE) Degradome-Seq Four degradome libraries were constructed by pooling RNA of the biological replicates used in sRNASeq and sequenced on the Illumina HiSeq 2500 platform^13^. The Degradome-Seq data were analyzed at LC Sciences (Houston, TX, USA) with CleaveLand4^46^) using all our experimentally identified sRNAs and the StNIB_v1 transcript sequences allowing for a maximum of three mismatches. All identified degradation targets were classified into 5 categories as previously described^46^. Category ‘0’ is defined as >1 raw read at the position, with abundance at a position equal to the maximum on the transcript, and with only one maximum on the transcript. Category ‘I’ is described as >1 raw read at the position, with abundance at the position equal to the maximum on the transcript, and more than one maximum position on the transcript. Category ‘II’ includes >1 raw read at the position and abundance at the position less than the maximum but higher than the median for the transcript. Category ‘III’ comprised the transcripts with >1 raw read at the position, and abundance at the position equal to or less than the median for the transcript. Category ‘IV’ comprised transcripts with one raw read at the cleavage position. Only categories with high confidence of cleavage (0, I, II, III) were considered for biological interpretation.

To evaluate the influence of sRNAs on mRNA abundance, we compared the expression of sRNAs with the expression of their target transcripts. All sRNAs and their targets, obtained by *in silico* prediction and Degradome-Seq were integrated with their expression data and used for the construction of regulatory networks in Cytoscape 3.4^13,47^.

### Proteomics analysis

For proteomics analysis, the second and third bottom leaves (2B, 3B) of mock- or PVY-inoculated plants of both genotypes were sampled at 4 dpi and pooled into 12 samples^13^. Plant material (100 mg) was powdered in liquid nitrogen and proteins were extracted from it with TRIzol mini-protocol^48^. Bradford assay^49^ was used to determine protein concentrations in the extracts, from which proteins (200 μg) were subjected to digestion and desalting^50^. Protein digest (0.5 µg) was loaded onto a Peptide ES-18 column (15 cm × 0.1 mm; 2.7 µm; Sigma-Aldrich, MO, USA) with a one-dimensional nano-flow LC system (UltiMate 3000, Thermo Fisher Scientific) coupled to an Orbitrap LTQ XL mass spectrometer (Thermo Fisher Scientific), operated in data-dependent mode. Peptides were eluted using a 60-min gradient from 5% to 80% acetonitrile/ 0.1% formic acid, with a controlled flow rate of 0.3 nL per min^50^.

The proteins were identified using the SEQUEST algorithm^51^ and Proteome Discoverer (v 1.3, Thermo Fisher Scientific). *In silico* peptide lists were generated with trypsin as the digestion enzyme, allowing for a maximum of three missed cleavages. Mass tolerance was set to 5 ppm for precursor ions and 0.8 Da for fragment ions. Peptides were identified by comparison to reference transcriptome StNIB_v1^35^. Additionally, a decoy database containing reversed sequences was used to estimate the false discovery rate (FDR). Only high confidence (FDR corrected p-value ≤ 0.01) peptide identifications with a minimum XCorr of 2.0, and proteins with at least two distinct peptides were considered as identified. For quantification, two approaches were used. In the first, spectral count information (Proteome Discoverer), missing values of unidentified proteins in the sample were imputed as half of the minimum protein expression value across all samples^52^. The second approach was the label-free quantification (LFQ) data matrix of MaxQuant software (v1.5.3.8)^53^. Differentially abundant proteins were determined using Student t-test (p < 0.05).

### Hormone measurements

For hormone measurements, the second bottom leaves (2B) of mock- or PVY-inoculated plants from both genotypes were sampled from 0–7 dpi in 4 replicates. Concentration of seven different plant hormones (ABA, GA3, OPDA, JA, IAA and SA) was determined by gas chromatography coupled with mass spectrometry (GC-MS). Tissue samples (~100 mg) were homogenized using a Tissue Lyser (Qiagen) and stainless steel beads (Qiagen) at −80 °C. A mixture of 1 ml of 100% methanol with 50 pmol of stable isotope-labelled internal standards was added to each sample. The samples were first heated (60 °C, 5 min) and then incubated at room temperature with occasional vortexing for 1 h. After centrifugation the methanolic phase was vacuum dried. The resulting residue was dissolved in combination of methanol (50 μl) and diethyl ether (200 μl). The samples were sonified (5 min) and centrifuged (5 min, 14,000 *g*). The particle-free supernatant was loaded to aminopropyl solid-phase extraction cartridges (Chromabond NH_2_; Macherey-Nagel GmbH, Düren, Germany). Each cartridge was washed twice with CHCl_3_:2-propanol (2:1, v/v, 250 μl) before the hormone-containing fraction was eluted with acidified diethyl ether (2% acetic acid, v/v, 400 μl). The eluates were transferred into 0.8 ml autosampler vials and dried. Prior to GC-MS analysis, the samples were derivatized with a 20 μl of a mix of acetone:methanol (9:1, v/v, 220 μ), diethyl ether (27 μl) and (trimethylsilyl)diazomethane solution (2.0 M in diethyl ether, 3 μl) and incubated at room temperature for 30 min. Settings for the GC-MS were as described previously^54^. For the determination of endogenous and stable isotope-labeled methylated acidic plant hormones, respectively, the following ion transitions were recorded: MeSA m/z 152 to m/z 120 and m/z 156 to m/z 124 for [^2^H_4_]-MeSA, retention time 6.75 ± 0.4 min; MeOPDA m/z 238 to m/z 163 and m/z 243 to m/z 168 for [^2^H_5_]-MeOPDA, retention time 10.00 ± 0.4 min; MeJA m/z 224 to m/z 151 and m/z 229 to m/z 154 for [^2^H_5_]-MeJA, retention time 11.27 ± 0.5 min; MeIAA m/z 189 to m/z 130 and m/z 191 to m/z 132 for [^2^H_2_]-MeIAA, retention time 13.34 ± 0.4 min; MeABA m/z 162 to m/z 133 and m/z 168 to m/z 139 for [^2^H_6_]-MeABA, retention time 15.78 ± 0.4 min; and MeGA m/z 136 to m/z 120 and m/z 138 to m/z 122 for [^2^H_2_]-MeGA, retention time 21.67 ± 0.6 min. The amounts of endogenous hormone contents were calculated from the signal ratio of the unlabeled over the stable isotope-containing mass fragment observed in the parallel measurements^13^. Significant changes for a set of hormones between treatment-genotype groups were determined by ANOVA followed by LSD post hoc analysis (FDR < 0.05) using the Agricolae R package^13^.

### Gas exchange and fluorescence measurements

Measurements of photosynthesis performance were taken between 9:00 and 11:00 AM on six plants of each treatment group (mock-inoculated Désirée, mock-inoculated NahG-Désirée, PVY-inoculated Désirée, PVY-inoculated NahG-Désirée), starting one day before inoculation (−1 dpi). The two bottom inoculated leaves (at 0, 1, 3, 4, 5, 7, 8 and 11 dpi) and two upper systemic leaves (at 1, 3, 4, 5, 7, 8 and 11 dpi) per plant were examined for each treatment group. Measurements were taken with a Li-6400 (LiCor, Lincoln, USA) measuring system equipped a 6400–40 Leaf Chamber Fluorometer. To perform measurements under the conditions that suit growing conditions, the chamber was mounted on a small tripod and positioned on a shelf of the growth chamber. At each measurement, the leaf was enclosed in the chamber and left to achieve a steady-state response, then a saturating light pulse was triggered to induce fluorescence, and in parallel, the gas-exchange data were stored. The data on net photosynthesis (P_n_), stomatal conductance (Cond), actual photochemical efficiency (Fv’/Fm’), potential photochemical activity (Fv/Fm), chlorophyll content (SPAD) and electron transport rate (ETR) of mock- and PVY-inoculated plants. Statistical model matrix was set to define contrasts as differences in mock normalized values between consecutive time points for each genotype^13^.

## Data Records

The complete Investigation has been deposited to FAIRDOMhub^13^. Microarray, sRNA-Seq and Degradome-Seq data can be accessed at the NCBI’s Gene Expression Omnibus repository^55–57^. Proteomics data are available via ProteomeXchange with identifier PXD015221^58^.

## Technical Validation

### RT-qPCR assays used in this study

qPCR assays: RT-qPCR assay targets for mRNA quantification are specified, together with their gene IDs, sequences of primers (Fw, Rw) and probe (P) and assay efficiencies. miRNA assays: TaqMan MicroRNA Assays, ordered according to the sRNA-Seq sequence of the selected miRNAs together with their IDs, mature miRNA sequence and efficiency of amplification^13^.

### Validation of microarray results by RT-qPCR

Microarray results were validated by quantitative real-time PCR (RT-qPCR). Eight biologically relevant genes were analyzed: for photosynthesis genes encoding chlorophyll a-b binding protein (CAB, CAB_NEW) and RuBisCO activase (RA); for defense response genes coding for three classes of β-1,3-glucanases (Glu-I, Glu-II, Glu-III) and pathogenesis-related protein1b (PR-1b); and for sugar metabolism granule-bound starch synthase I (GBSSI) and cell wall invertase (INV) genes. Pearson correlation between the results of both methods (microarrays, RT-qPCR) was high (0.74; Table 1).

**Table 1:** Validation of microarray results by RT-qPCR.

		Desiree										Desiree-NahG									
		dpi1		dpi3		dpi4		dpi5		dpi7		dpi1		dpi3		dpi4		dpi5		dpi7	
		μarray	qPCR	μarray	qPCR	μarray	qPCR	μarray	qPCR	μarray	qPCR	μarray	qPCR	μarray	qPCR	μarray	qPCR	μarray	qPCR	μarray	qPCR
**CAB**	cSTD1O21THB	**1.4**	0.3	0.3	−0.4	**1.1**	1.3	−0.5	0.4	0.1	2.1	1	−0.8	0.1	0	0	−0.6	0.2	1.3	−0.1	0.6
	MICRO.331.C90	0.1		0		0.5		0.1		−0.1		−0.1		−0.1		0		0.2		0	
	MICRO.331.C84	1.2		0.6		0.6		−0.6		−0.6		−1.1		−0.5		**−1.4**		−0.7		−1	
	MICRO.331.C81	**3.5**		**2.9**		**2.7**		**3.3**		**3**		−0.8		−0.5		0.6		0.5		−0.8	
	MICRO.331.C62	**0.9**		**0.8**		**0.8**		**0.8**		0.2		**−0.9**		0.1		0		0.2		−0.3	
	MICRO.331.C60	0.2		−0.1		**1.8**		−0.1		−0.3		−0.4		0.2		−0.6		0.5		0	
	MICRO.331.C89	1.1		−0.4		**2.3**		−0.9		0.2		−1.4		1.2		0.3		1.6		0.7	
	MICRO.331.C79	0.6		−0.1		**2.2**		−0.5		−0.4		−0.3		−0.2		−1.8		0.4		0.1	
	MICRO.331.C76	0.9		−0.2		**1.4**		−0.6		−0.3		0.7		−0.1		−0.1		0.3		0.3	
	MICRO.331.C27	1.7		0.3		**2.5**		−0.1		1.3		−1.7		0.8		1		1.3		0.4	
	MICRO.331.C12	**0.8**		**0.7**		0.5		0.1		0.1		**0.8**		0		0.4		0.4		−0.4	
	MICRO.331.C9	0.5		0.1		1.8		−0.1		0.1		0.3		0.5		0.4		0.8		0.3	
**INV**	STMIM75TV	−0.6	−0.6	0.3	0	0	−1	0.1	−1.7	0.5	1	0.4	1.1	−0.5	**1**	**1.4**	1.1	**1.5**	2.2	**1.8**	1.7
**GBSS1**	MICRO.920.C5	0.2	0.4	−0.3	0.4	0.3	0.9	**−1.4**	−0.8	−0.3	0.3	−0.5	−2.1	0	−0.3	−1.3	**−1.5**	−0.3	−0.3	−0.5	−0.1
	MICRO.920.C2	0.3		−0.4		0.7		−1.5		0		−0.4		0.1		−1.4		−0.2		−0.4	
**Glu-I**	MICRO.2526.C3	0.2	**1.5**	−0.1	−0.2	0	**1.8**	−0.2	−0.6	0.1	1.8	**0.3**	4.5	0.2	0.6	0.3	2.8	0.1	2.2	**0.4**	3
**Glu-II**	MICRO.2286.C42	−0.6	−0.3	−0.7	−0.6	0.1	0.7	−0.6	−1.7	−0.2	−1.2	0.4	−0.2	−0.7	2.4	−0.8	−1.4	2.1	2.5	3.7	4.3
	MICRO.2286.C28	0		−0.2		0.5		0.1		**0.7**		**−0.7**		0.5		0		**0.6**		−0.5	
	MICRO.2286.C15	−0.3		−0.7		0.2		−0.9		−0.1		0.7		−0.8		−0.9		**1.8**		**3.9**	
	MICRO.2286.C1	0		0		0		0.1		0.2		0		**0.2**		**0.4**		**0.3**		−0.1	
**Glu-III**	MICRO.6187.C2	−0.4	−0.5	−0.3	−0.5	0.4	0.5	0.2	−1	0.4	−0.4	−0.3	−0.2	−1.1	−0.5	1.4	0.5	**3.6**	**3.1**	**3.9**	4.6
	MICRO.6187.C1	−0.4		−0.2		0.9		−0.1		0.4		0.1		−1.8		0.6		**3.8**		**4.5**	
**PR1b**	MICRO.5426.C4	−0.2	−0.6	−1.7	−0.8	0.5	1.3	−1.3	−1.6	1.3	1.5	−1.2	−3.8	−2.3	−2	−0.4	0	**4.4**	**5.6**	**5.9**	**5.4**
**RA**	MICRO.4141.C1	0.2	−0.6	−0.4	**−1**	−0.4	−0.6	**−1.3**	**−1.5**	**−1.7**	**−1.8**	0.6	−0.9	−0.3	−0.4	−1	**−0.9**	−0.4	0.5	−0.1	0.8

Microarray results were validated by analyzing eight biologically relevant genes coding for proteins involved in photosynthesis (chlorophyll a-b binding protein: CAB and RuBisCO activase: RA), defense response (β-1,3-glucanase of three classes: Glu-I, Glu-II, Glu-III and pathogenesis-related protein1b: PR-1b), and sugar metabolism (granule bound starch synthase I: GBSSI and INV: cell wall invertase) by quantitative real-time PCR. Expression values were log2 transformed, and a fold-change difference (log2FC) was calculated for PVY^NTN^ versus mock in cv. Désirée and NahG-Désirée at 1, 3, 4, 5 and 7 dpi are shown in the table. Statistically significant values (p < 0.05) are marked with bold.

### miRNA stem-loop RT-qPCR for sRNA-Seq data validation

**Fig. 5:**
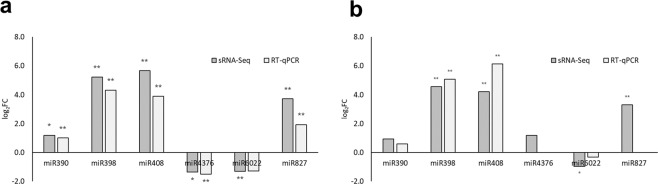
Validation of sRNA-Seq results by stem-loop RT-qPCR. The log_2_ ratios of expression of six miRNAs in PVY^NTN^-infected samples versus mock-inoculated samples of potato cv. (**a**) Désirée or (**b**) NahG-Désirée as determined by sRNA-Seq (dark grey) or RT-qPCR (light grey). The expressions of miR827 and miR4376 in NahG-Désirée samples were under the limit of quantification. Asterisks indicate statistically significant changes as determined by Student t-test (n = 3; **p-value < 0.05; *p-value < 0.1). miR390 – stu-miR390-5p; miR398 – stu-miR398a-5p; miR408 – stu-miR408b-5p; miR4376 – stu-miR4376-5p.1; miR6022 – stu-miR6022-3p; miR827 – stu-miR827-5p. See Methods for details of the experimental procedure.

sRNA expression results obtained by sRNA-seq were validated by stem-loop RT-qPCR. For validation experiments, the same RNA samples as used for sRNA-Seq were analyzed. As previously described in Križnik *et al*.^17^, using RT-qPCR analysis we validated all sRNA-Seq differential expression results except in cases were concentrations of miRNAs were below the limit of the quantification. Pearson correlation between the results of both methods (sRNA-Seq, stem-loop RT-qPCR) was very high (0.92; Fig. 5).

### Degradome-Seq for sRNA target validation

Degradome-Seq was used to validate miRNA-target pairs *in silico* predicted by the psRNATarget tool^44^. Degradome-Seq experimentally identified 3,015 unique sRNA-target cleavage pairs, between 1,042 unique sRNAs and 1,663 unique target mRNAs. psRNATarget tool predicted 36,750 different unique sRNA-target pairs between 1,960 potato sRNAs and 15,000 non-redundant potato mRNA targets. The majority of predicted interactions (29,336; ~80%) were predicted as cleavage type interactions.

**Fig. 6:**
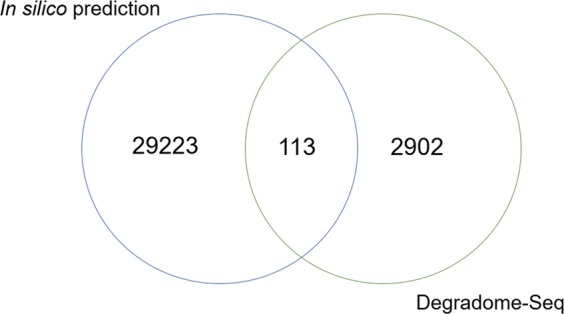
Intersection between Degradome-Seq and *in silico* predictions of sRNA-target cleavage interactions.

Comparison between both by *in silico* and experimental target prediction revealed 113 commonly identified sRNA-target pairs (Fig. 6). Among them, 24 were miRNA-target pairs and 89 were phasiRNA-mRNA pairs. Most verified miRNA-target interactions resulting in mRNA degradation were highly conserved miR160-*ARF10*/*ARF17*, miR172-*APETALA2*, miR319-*TCP*, miR393-*TIR1*, miR396-*GRF* modules. The limited number of sRNA-target pairs predicted with both approaches are most probably related to differences in prediction parameters employed within *in silico* or Degradome-Seq analysis (i.e. Cleaveland pipeline^46^). Hence, negative expression correlations between sRNAs and their target genes were used to biologically characterize the identified interactions.

## Data Availability

The code used for analysis of microarray data and differential expression for microarray sRNAomics and hormonomics data is available at FAIRDOMHub^13^ for project MOA on the corresponding Experimental Assay subsections.
